# AEG-1 targeting for inhibiting inflammation: potential anti-HCC strategy

**DOI:** 10.18632/oncotarget.26602

**Published:** 2019-01-18

**Authors:** Saranya Chidambaranathan Reghupaty, Rachel Mendoza, Devanand Sarkar

**Affiliations:** Department of Human and Molecular Genetics, Massey Cancer Center, VCU Institute of Molecular Medicine (VIMM), Virginia Commonwealth University, Richmond, VA, USA

**Keywords:** AEG-1, HCC, inflammation, macrophages, NF-kappaB

Hepatocellular carcinoma (HCC) is a primary liver cancer arising from hepatocytes and accounts for approximately 80% of liver cancers. The main risk factors for HCC are viral infections, such as hepatitis B virus (HBV) or hepatitis C virus (HCV), alcoholism, and obesity-induced non-alcoholic fatty liver disease (NAFLD), all of which lead to chronic inflammation. Understanding the mechanism of the chronic inflammatory process facilitates development of novel strategies to counteract this fatal disease.

Astrocyte elevated gene-1 (AEG-1) is an oncogene that is over-expressed in many cancers including HCC. Forced over-expression of AEG-1 both *in vitro* and *in vivo* increases all cancer hallmarks, such as proliferation, invasion, migration, angiogenesis, metastasis and chemo-resistance whereas the knock-down of AEG-1 has the reverse effect [1]. AEG-1 is a scaffold protein that mediates its function by protein-protein and protein-RNA interaction resulting in the activation of key oncogenic signaling pathways, such as PI3K/Akt, EGFR/MAPK, Wnt/β-catenin and NF-κB. Knocking out AEG-1 in mice render them resistant to experimental HCC [2] and AEG-1 inhibitory strategies show profound efficacy in inhibiting HCC in mouse models [3].

AEG-1 plays a fundamental role in activating the transcription factor NF-κB [4] which regulates the transcription of pro-inflammatory cytokines and thereby functions as a master regulator of inflammation. Because chronic inflammation is the underlying event in hepatocarcinogenesis irrespective of etiology, NF-κB activation plays a key role in this process [5]. As such, AEG-1-/- mouse shows profound inhibition of inflammation resulting in resistance to HCC [2]. Indeed, AEG-1-mediated regulation of pro-inflammatory signaling pathway also functions as an underlying event in gastric cancer and rheumatoid arthritis [6, 7].

Liver-resident macrophages (Kupffer cells) play a vital role in creating a pro-inflammatory and pro-tumorigenic microenvironment for HCC development [8]. When hepatocytes are injured, e.g., by viral infection or fat accumulation, the damaged hepatocytes release cytokines that stimulate activation of NF-κB in Kupffer cells resulting in release of cytokines that activate oncogenic signaling pathways in the hepatocytes resulting in transformation (Figure 1, left panel). In addition, there is also infiltration of monocyte-derived macrophages into the liver further contributing to the inflammatory process. During tumorigenesis, the undifferentiated monocyte-derived macrophages are activated by classical activation into M1 macrophages that are pro-inflammatory or by alternative activation to M2 macrophages that are pro-tumorigenic. In a chronic inflammatory disease like HCC, there is a continuous flux of M1 and M2 macrophages contributing to the inflammatory tumorigenesis process.

**Figure 1:**
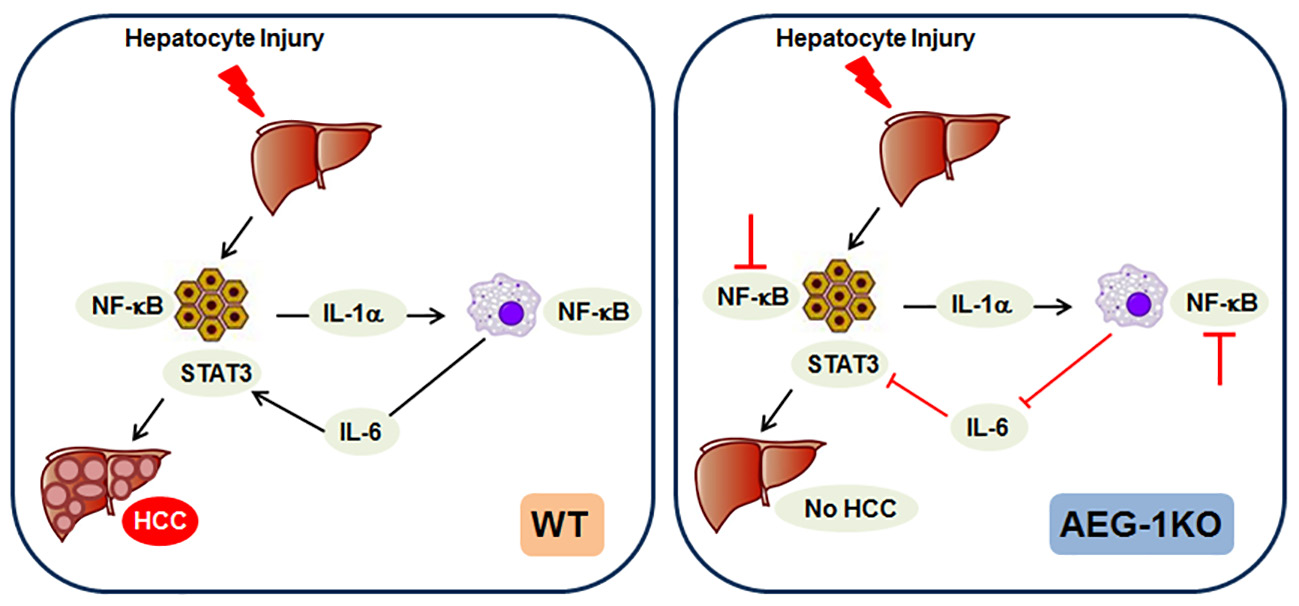
Cartoon showing the mechanism by which AEG-1 regulates the inflammatory process and HCC development **Left panel.** In Wild-type (WT) mice injury to hepatocytes activates NF-κB and IL-1 production that activates NF-κB and IL-6 production in the macrophages. IL-6 activates oncogenic STAT3 in hepatocytes facilitating transformation and development of HCC. **Right panel.** In the absence of AEG-1 (AEG-1KO), NF-κB activation is inhibited in hepatocytes and macrophages, thereby abrogating the inflammatory signaling and HCC development. Adapted from Reference 2.

It was documented that AEG-1 expression in the macrophages is significantly higher than that in hepatocytes indicating that AEG-1 might regulate macrophage function [2]. Overexpression of AEG-1 in macrophages promoted invasion by hypopharyngeal cancer cells in a co-culture system, and macrophages in the hypopharyangeal tumor tissue expressed more AEG-1 than macrophages in adjacent non-tumor tissue [9]. Employing conditional knockout mouse models, Robertson *et al*. demonstrate that while global deficiency of AEG-1 in AEG-1-/- mice completely abrogated experimental HCC, hepatocyte-specific AEG-1 deficiency (AEG-1^ΔHEP^) led to only an attenuation (and not complete abrogation) of HCC, while myeloid cell-specific AEG-1 deficiency (AEG-1^ΔMAC^) led to complete abrogation of HCC, indicating that AEG-1 plays a key role in initial macrophage activation that is crucial for hepatocyte transformation [10]. *In vitro* studies documented that AEG-1-/- macrophages were resistant to either M1 or M2 differentiation with significant inhibition in migration, endothelial adhesion and efferocytosis activity, indicating that AEG-1 ablation renders macrophages functionally anergic [10]. These findings indicate that AEG-1 activation of NF-κB in both hepatocytes and Kupffer cells contributes to HCC development (Figure 1). Thus AEG-1 targeting in both HCC cells and HCC-associated macrophages might be an efficient therapeutic strategy for HCC. A hepatocyte-targeted nanoparticle delivering AEG-1 siRNA shows strong therapeutic efficacy against HCC [3] and might be combined with a macrophage-targeted nanoparticle to impart a robust and lasting effect.

## References

[1] Sarkar D (2013). Adv Cancer Res.

[2] Robertson CL (2014). Cancer Res.

[3] Rajasekaran D (2015). Bioconjug Chem.

[4] Sarkar D (2008). Cancer Res.

[5] Pikarsky E (2004). Nature.

[6] Li G (2014). Cancer Res.

[7] Hong R (2017). Tissue Cell.

[8] Krenkel O (2017). Nat Rev Immunol.

[9] Liu X (2016). Oncotarget.

[10] Robertson CL (2018). Cancer Res.

